# Classification of rhythmic locomotor patterns in electromyographic signals using fuzzy sets

**DOI:** 10.1186/1743-0003-8-65

**Published:** 2011-12-08

**Authors:** Timothy A Thrasher, John S Ward, Stanley Fisher

**Affiliations:** 1 Dept of Health and Human Performance, (Center) for Neuromotor and Biomechanics Research, University of Houston, Houston, TX, USA; 2 The Methodist Neurological Institute, Houston, TX, USA

**Keywords:** Surface electromyography, gait, central pattern generator, fuzzy analysis

## Abstract

**Background:**

Locomotor control is accomplished by a complex integration of neural mechanisms including a central pattern generator, spinal reflexes and supraspinal control centres. Patterns of muscle activation during walking exhibit an underlying structure in which groups of muscles seem to activate in united bursts. Presented here is a statistical approach for analyzing Surface Electromyography (SEMG) data with the goal of classifying rhythmic "burst" patterns that are consistent with a central pattern generator model of locomotor control.

**Methods:**

A fuzzy model of rhythmic locomotor patterns was optimized and evaluated using SEMG data from a convenience sample of four able-bodied individuals. As well, two subjects with pathological gait participated: one with Parkinson's Disease, and one with incomplete spinal cord injury. Subjects walked overground and on a treadmill while SEMG was recorded from major muscles of the lower extremities. The model was fit to half of the recorded data using non-linear optimization and validated against the other half of the data. The coefficient of determination, R^2^, was used to interpret the model's goodness of fit.

**Results:**

Using four fuzzy burst patterns, the model was able to explain approximately 70-83% of the variance in muscle activation during treadmill gait and 74% during overground gait. When five burst functions were used, one function was found to be redundant. The model explained 81-83% of the variance in the Parkinsonian gait, and only 46-59% of the variance in spinal cord injured gait.

**Conclusions:**

The analytical approach proposed in this article is a novel way to interpret multichannel SEMG signals by reducing the data into basic rhythmic patterns. This can help us better understand the role of rhythmic patterns in locomotor control.

## Background

During gait, the Central Nervous System (CNS) activates the muscles of the lower extremities in rhythmic patterns that can be measured by surface electromyography (SEMG). These signals are not precisely periodic; they naturally vary from stride to stride due to responses to environmental stimuli and a number of complex mechanisms in the CNS that are not well understood. SEMG is often used in the study of the motor control of normal and pathological gait, because it contains important information about the timing and intensity of muscle commands that originate in the CNS [1]. There have been several attempts to statistically classify locomotor patterns from SEMG data, however the majority of these approaches are *a posteriori *and identify patterns without regard for physiological theory. Here, we propose a new *a priori *analytical method involving fuzzy systems that is designed to classify rhythmic locomotor patterns in SEMG waveforms that fit a rudimentary model of open-loop Central Pattern Generator (CPG) control.

Interpretation of SEMG during gait is particularly challenging due to the complexity of the myoelectric signals, which are stochastic in nature and represent an interference pattern from multiple motor units. Furthermore, SEMG data are usually multi-dimensional and involve significant measurement error (noise) that can only be partially discriminated from true signal using filtering techniques [2]. A number of statistical techniques have been proposed to deal with the high dimensionality and uncertainty that is inherent to SEMG data [3,4]. Jansen et al. [5] used a hierarchical clustering procedure to classify different muscle patterns observed in gait, from which they were able to draw inferences about different walking strategies. Intra-class correlation coefficients have been used to identify characteristics of different patient populations [6]. Factor analysis has been used to capture the underlying correlations between muscles, which has led to a deeper understanding of how locomotor patterns are organized [7]. These advanced analytical approaches can contribute to a better understanding of the underlying neural mechanisms that control muscle activity during gait. However, these approaches are *a posteriori *and lead to identification of patterns independent of physiological theory. The method proposed here is built upon the specific theory of a CPG that open-loop control of locomotion using simplified, pre-programmed muscle commands.

The idea that human locomotion is driven by oscillating neural circuits located in the spinal cord has been advanced for decades [8]. These circuits, known as the CPG, provide rhythmic "bursts" of muscle activation signals that form the basis of locomotor control [9-11]. By analyzing the basic pattern of SEMG signals as well as the variability that occurs over multiple strides, we can gain valuable insight into the function of the CPG and its role in human locomotor control.

One of the most important challenges in gait analysis is to determine if a set of recorded signals represents normal gait or if it contains particular signatures of pathological gait. It is often desirable to compare one set of SEMG waveforms to another in order to determine if a subject's gait exhibits abnormal behavior, if an intervention was successful, or if walking under different conditions involves different muscle activation patterns. Some researchers have developed mathematical indices that quantify certain features of dynamic EMG waveforms for the purpose of quantifying impairment [12,13] or to evaluate stride-to-stride variability [14].

Many neurological disorders are associated with increased variability of gait [1,5,9,15]. This is due to errors in locomotor control caused by dysfunction of specific areas in the CNS. It is conceivable that some CNS disorders may actually reduce the amount of variability, due to a decrease in anticipatory control (supraspinal), a decrease in environmental interaction (spinal reflexes) and a relative increase in self-generated oscillatory commands form the spinal CPG. For example, Miller et al. [14] observed reduced timing variability of the gastrocnemius muscle in Parkinsonian gait. This is an interesting finding that suggests there may be other characteristics of pathological gait that produce abnormally invariant muscle activation signals.

This article describes a combined fuzzy and statistical approach that first classifies basic muscle activation patterns during different phases of the gait cycle, and then evaluates the degree to which recorded muscle signals are consistent with a rudimentary CPG model of locomotor control. This approach is unique in that it enables an estimate of how much of the variability in muscle activity in gait is due to recurring basic patterns and how much is due to error and non-rhythmic sources of control (i.e., anticipatory adjustments, aberrant reflexes, measurement error, etc.).

## Methods

### Subjects

SEMG recordings were collected from four able-bodied (AB) individuals with no neurological conditions, as well as one individual with Parkinson's Disease (PD) and one individual with incomplete Spinal Cord Injury (SCI). Descriptive data of the six subjects is provided in Table 1. PD subjects were classified according to the Hoehn & Yahr scale [16], and SCI subjects were classified according to the American Spinal Injury Association (ASIA) Impairment Scale [17]. PD is a neurological disorder in which the supraspinal centers are believed to generate erroneous signals for locomotion [18]. SCI was included as a case in which the pathways between supraspinal centers and spinal circuits are impaired. We expected to find abnormal features in the SEMG of both pathological subjects.

**Table 1:** Details of subjects

Subject	Group	Age	Gender	Disease/injury duration	Clinical classification	Walking speed (m/s)
1	AB	25	F	-	-	0.714
2	AB	22	F	-	-	0.667
3	AB	24	F	-	-	0.690
4	AB	32	M	-	-	0.769
5	PD	59	M	8 years	HAY 2^a^	0.625
6	SCI	42	M	3 years	T10, AIS C^b^	0.143

^a ^Hoehn & Yahr scale [16].

^b ^American Spinal Injury Association (ASIA) Impairment Scale [17].

### Instrumentation and protocol

Each subject was instrumented with an 8-channel SEMG system (Biometrics DataLOG, Biometrics Ltd, Ladysmith, VA, USA). Eight electrodes were carefully placed over the muscle belly of the following muscles bilaterally: vastus lateralis (VL), long head of biceps femoris (BF), tibialis anterior (TA) and gastrocnemius lateralis (LG). These particular muscles were selected as a representative set of the major actuators during gait [5]. The skin was cleaned and lightly abraded before the electrodes were attached with double-sided adhesive tape. SEMG signals were amplified, filtered (bandpass: 15 - 450 Hz), and recorded at 2000 Hz. A foot switch was placed in the right shoe directly under the heel to detect initial foot contact, which was used to mark the beginning and end of each gait cycle.

Each subject performed two trials of overground walking (OG) for a distance of 10 m. Then each subject performed two trials of treadmill walking (TM) for a duration of 30 s. TM speed was set to the average walking speed of the subject's OG trials. The first trial of each set was used as training data for optimizing the model. The second trial was used to validate the model.

After recording, SEMG signals were rectified and filtered using a low-pass Butterworth filter with a cut-off frequency of 10 Hz, which is considered sufficient for noise removal without loss of signal [2]. All signals were then separated into individual gait cycles marked by right foot contact and time-normalized relative to the gait cycle using cubic spline interpolation of 100 evenly spaced points in time (0 to 99% of the gait cycle). All data processing was performed using Matlab software (The Mathworks, Inc., Natick, MA, USA).

### Algorithm

The rectified and filtered SEMG signals were coded according to fuzzy sets [3,19]. A set of *n *Gaussian membership functions were used to represent specific bursts of muscle activity during the gait cycle. These are described by Equation 1. Gaussian functions represent a basic "burst" pattern and have been used previously to decompose SEMG data [20].

(1) $$b_{i}\left(t\right)=\frac{1}{\sigma_{i}\sqrt{2\pi }}e^{-{\left(t-\tau_{i}\right)}^{2}∕\left(2{\sigma_{i}}^{2}\right)}$$

**Figure 1:**
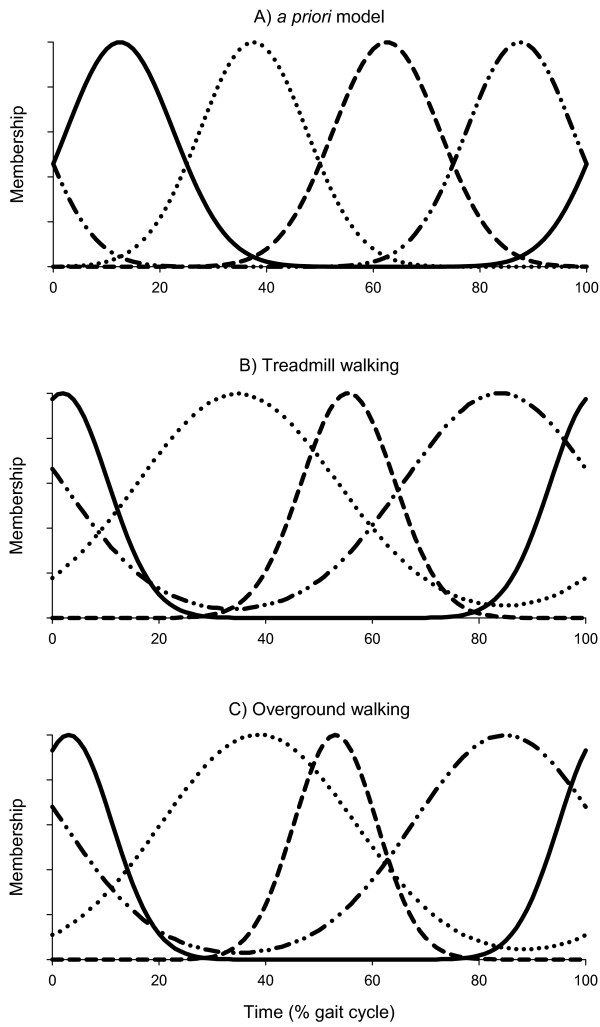
**Fuzzy models**. Burst functions representing four synergistic patterns of muscle activity during the gait cycle. A) Arbitrary bursts covering the gait cycle. B) Optimized with respect to overground walking data. C) Optimized with respect to treadmill walking data.

Where *b_i_(t) *is the *i*th burst function, τ*_i _*is the time of maximum value, and σ*_i _*is the standard deviation. The values of τ*_i _*and σ*_i _*were initially selected *a priori *to provide good coverage of the gait cycle. τ*_i _*were equally spaced throughout the gait cycle, and σ*_i _*were all equal to 10% of the gait cycle. Figure 1A illustrates the burst functions for n = 4, and the initial model parameters can be expressed as the following vectors.

$$\tau =\left\{\begin{array}{c} 12.5\\ 37.5\\ 62.5\\ 87.5 \end{array}\right\}\text{ and }\sigma =\left\{\begin{array}{c} 10\\ 10\\ 10\\ 10 \end{array}\right\}$$

Each SEMG signal was treated as a weighted sum of the burst functions. Our model is described in Equation (2).

(2) $$Y_{j}\left(t\right)=w_{j1}\cdot b_{1}\left(t\right)+\cdots +w_{jn}\cdot b_{n}\left(t\right)$$

Where *Y_j_(t) *is the SEMG signal of the *j*th muscle and *w_ji _*is the weighting coefficient for the *j*th muscle and the *i*th burst function. *n *is the number of burst functions. The weighting coefficients were determined by fitting the model to the recorded SEMG data using a least-squares linear regression (Matlab function lscov). Each muscle was therefore represented by a single n-element vector of phase coefficients, resulting in a major reduction in the information density of each signal. Each SEMG signal could then be reconstructed using n coefficients, creating a basic underlying pattern of muscle activation during the gait cycle. These coefficients can be interpreted as the pre-programmed muscle activation patterns that are dispensed by the CPG at the different phases of the gait cycle.

The model was optimized by finding the values of *τ_i _*and *σ_i _*that produced the best fit. A Nelder-Mead simplex direct search algorithm (Matlab function fminsearch) was used to find the burst function parameters that maximized the goodness of fit, *R^2^*, between the training data and the model output. We interpreted *R^2 ^*as the proportion of the variance in the SEMG signals that is explained by the model.

## Results

### Testing

**Figure 2:**
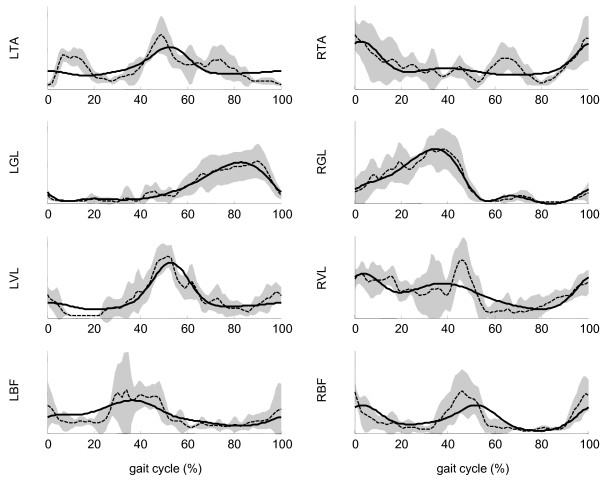
**SEMG data**. Representative sample of SEMG signals for the 8 muscles observed in this study (AB subject 3 walking overground). Ensemble average SEMG are shown as dashed lines. Grey area represents the mean plus and minus one standard deviation. The solid line is the model output.

A 4-burst model was fit to the treadmill walking data and the overground walking data separately. Four bursts were initially chosen, because models of the CPG typically consist of four synergies corresponding to a flexor pattern and an extensor pattern on each side of the body [8]. As show in Figure 1, the burst function profile of these two models differed only slightly. Figure 2 shows the SEMG data from one of the validation trials of overground walking, and the model estimation of the SEMG profiles for all eight muscles.

**Figure 3:**
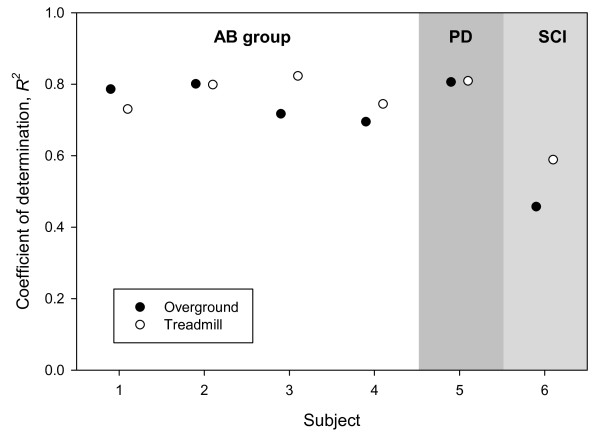
**Goodness of fit**. Goodness of fit of the models with respect to the validation data.

Following optimization of the model, a separate *R^2 ^*was calculated for each subject under each walking condition (OG and TM) using the validation data. Figure 3 summarizes the *R^2 ^*values under each walking condition. This represents to what extent the fuzzy model accounts for the variance of all SEMG signals of the validation walking trial.

Initially, the model was designed with n = 4 burst functions. We tested for improved model performance by increasing the number of bursts from four to five. The best fit solution resulted in two functions with identical parameters values for τ and σ. In other words, the 5-burst model degenerated to a 4-burst model. The fifth burst was redundant and provided no improvement to the fit of the model.

## Discussion

The approach presented in this article represents a form of fuzzy coding of muscle activation signals that can be used to determine an underlying temporal pattern of SEMG signals during gait. The basic structure of the predictor model consists of four overlapping Gaussian membership functions distributed across the gait cycle. This model is based on general theory of CPG control of locomotion. The Gaussian membership functions representing pre-programmed bursts from the CPG were optimized according to a set of training data and then tested against a set of validation data. Four burst functions were sufficient; when a fifth burst was added, the model degenerated into a four-burst model during optimization.

The model assumes that the CPG produces periodic signals that are exactly the same for every stride. From this we may conclude that all stride-to-stride variability is due to mechanisms other than the CPG, i.e., anticipatory adjustments from supraspinal centers, reflex responses to external perturbations, etc. In normal gait, the model was able to account for 70-84% of the variance in SEMG throughout the gait cycle. Similar results were found for the subject with Parkinson's Disease. The model was not able to account for the SEMG of the SCI subject very well, likely due to a lack of coordination and high stride-to-stride variability.

Our statistical approach differs significantly from other methods of interpreting SEMG data during gait. Many SEMG analyses focus on the ensemble average of all strides and do not take into account variability [3,21]. In our analysis, the stride-to-stride variability was essential in determining the goodness of fit of the fuzzy CPG model. Ivanenko et al. [7] used factor analysis to find common waveforms that were shared by multiple muscles. These waveforms are analogous to the Gaussian membership functions that we use in our model, however they are more complex in shape. They were able to account for roughly 80% of the variance in normal gait, which is similar to our results [22].

There are some special considerations when using the analytical method described in this article. First, *R^2 ^*is very sensitive to measurement error, so great care should be taken to ensure that electrodes are placed correctly and securely. The calculation of *R^2 ^*is based on an estimation of variance using sums of squares. Considering the *n*-channel SEMG data as a set of points in *n*-dimensional space the sums of squares are based on Euclidean distances, whereby each dependent variable has equal weight. This may not always be appropriate. For example, if recordings are taken from the soleus and both heads of gastrocnemius, the triceps surae will contribute three times as much to the sum of squares as other muscle groups that are recorded individually.

## Conclusions

The analytical approach proposed in this article is a novel way to interpret multichannel SEMG signals by reducing the data into basic rhythmic patterns. This can help us better understand the role of rhythmic patterns in locomotor control, and provide insight about certain forms of pathological gait.

## Competing interests

The authors declare that they have no competing interests.

## Authors' contributions

TAT conceived the basis for the study, designed the methodology and carried out the data processing and statistical analyses. JSW collected the bulk of the data and participated in the data processing. SF contributed to the design of the study, recruitment of subjects, and analysis of data. All authors have read and approved the final version of this article.

## References

[B1] Frigo C Crenna P Multichannel SEMG in clinical gait analysis: a review and state-of-the-art Clin Biomech 2009 24 3 236 45 10.1016/j.clinbiomech.2008.07.012 18995937

[B2] Shiavi R Frigo C Pedotti A Electromyographic signals during gait: criteria for envelope filtering and number of strides Med Biol Eng Comput 1998 36 2 171 8 10.1007/BF02510739 9684456

[B3] Chau T A review of analytical techniques for gait data. Part 1: Fuzzy, statistical and fractal methods Gait Posture 2001 13 1 49 66 10.1016/s0966-6362(00)00094-1 11166554

[B4] Shan G Visentin P Schultz A Multidimensional Signal Analysis as a Means of Better Understanding Factors Associated with Repetitive Use in Violin Performance Med Probl Perform Art 2004 19 3 129 139

[B5] Jansen BH Miller VH Mavrofrides DC Stegink Jansen CW Multidimensional EMG-based assessment of walking dynamics IEEE T Neural Syst Rehabil Eng 2003 11 3 294 300 10.1109/TNSRE.2003.816865 14518794

[B6] Duhamel A Bourriez JL Devos P Krystkowiak P Destée A Derambure P Defebvre L Statistical tools for clinical gait analysis Gait Posture 2004 20 2 204 12 10.1016/j.gaitpost.2003.09.010 15336292

[B7] Ivanenko YP Grasso R Zago M Molinari M Scivoletto G Castellano V Macellari V Lacquaniti F Temporal components of the motor patterns expressed by the human spinal cord reflect foot kinematics J Neurophysiol 2003 90 5 3555 3565 10.1152/jn.00223.2003 12853436

[B8] Pinter MM Gait after spinal cord injury and the central pattern generator for locomotion Spinal Cord 1999 37 8 531 537 10.1038/sj.sc.3100886 10455527

[B9] Ivanenko YP Cappellini G Dominici N Poppele RE Lacquaniti F Coordination of locomotion with voluntary movements in humans J Neurosci 2005 25 31 7238 7253 10.1523/JNEUROSCI.1327-05.2005 PMC6725226 16079406

[B10] Ivanenko YP Poppele RE Lacquaniti F Distributed neural networks for controlling human locomotion: lessons from normal and SCI subjects Brain Res Bull 2009 78 1 13 21 10.1016/j.brainresbull.2008.03.018 19070781

[B11] Gallarda BW Sharpee TO Pfaff SL Alaynick WA Defining rhythmic locomotor burst patterns using a continuous wavelet transform Ann NY Acad Sci 2010 1198 133 139 10.1111/j.1749-6632.2010.05437.x PMC3334338 20536927

[B12] Chester V Using waveform analyses to develop pediatric gait indices Exercise Sport Sci R 2009 37 4 211 7 10.1097/JES.0b013e3181b7b879 19955871

[B13] Fung J Barbeau H A dynamic EMG profile index to quantify muscular activation disorder in spastic paretic gait Electroen Clin Neuro 1989 73 3 233 44 10.1016/0013-4694(89)90124-7 2475328

[B14] Miller RA Thaut MH Mcintosh GC Rice RR Components of EMG symmetry and variability in parkinsonian and healthy elderly gait Electroen Clin Neuro 1996 4 1 7 10.1016/0013-4694(95)00209-x 8625872

[B15] Delval A Salleron J Bourriez J-L Bleuse S Moreau C Krystkowiak P Defebvre L Devos P Duhamel A Kinematic angular parameters in PD: reliability of joint angle curves and comparison with healthy subjects Gait Posture 2008 28 3 495 501 10.1016/j.gaitpost.2008.03.003 18434159

[B16] Hoehn MM Yahr MD Parkinsonism: onset, progression and mortality Neurology 1967 17 5 427 442 10.1212/wnl.17.5.427 6067254

[B17] Maynard FM Bracken MB Creasey GJFD Donovan WH Ducker TB Garber SL Marino RJ Stover SL Tator CH Waters RL Wilberger JE Young W International Standards for Neurological and Functional Classification of Spinal Cord Injury Spinal Cord 1997 35 5 266 274 10.1038/sj.sc.3100432 9160449

[B18] Ferrarin M Carpinella I Rabuffetti M Rizzone M Lopiano L Unilateral and Bilateral Subthalamic Nucleus Stimulation in Parkinson's Disease: Effects on EMG Signals of Lower Limb Muscles During Walking IEEE T Neural Syst Rehabil Eng 2007 15 2 182 189 10.1109/TNSRE.2007.897000 17601187

[B19] Bouilland S Loslever P Multiple correspondence analysis of biomechanical signals characterized through fuzzy histograms J Biomech 1998 31 7 663 6 10.1016/s0021-9290(98)00054-2 9796689

[B20] Chen J-JJ Shiavi R Temporal feature extraction and clustering analysis of electromyographic linear envelopes in gait studies IEEE T Biomed Eng 1990 37 3 295 302 10.1109/10.52330 2184121

[B21] Chau T Young S Redekop S Managing variability in the summary and comparison of gait data J NeuroEng Rehabil 2005 20 2 22 10.1186/1743-0003-2-22 PMC1208939 16053523

[B22] Ivanenko YP Poppele RE Lacquaniti F Five basic muscle activation patterns account for muscle activity during human locomotion J Physiol 2004 556 Pt 1 267 82 10.1113/jphysiol.2003.057174 PMC1664897 14724214

